# How Guilt Drives Emotional Exhaustion in Work–Pet Family Conflict

**DOI:** 10.3390/ani14233503

**Published:** 2024-12-04

**Authors:** Ana Junça-Silva

**Affiliations:** Business Research Unit, ISCTE—Instituto Universitário de Lisboa, 1649-026 Lisboa, Portugal; analjsilva@gmail.com

**Keywords:** work–pet family conflict, work–life conflict, emotional exhaustion, guilt

## Abstract

Work–pet family conflict has emerged as a novel form of work–life conflict, reflecting the increasingly significant role that pets play in modern families. Guided by role theory, work–pet family conflict is anticipated to produce outcomes similar to those of traditional work–life conflict. Accordingly, we developed a conceptual model to examine how work–pet family conflict affects employees’ emotional exhaustion. Drawing on role theory, we tested whether the experience of guilt serves as an affective mechanism linking work–pet family conflict to emotional exhaustion. Data were collected from 356 pet owners to empirically test the model. The results revealed a significant relationship between work–pet family conflict and emotional exhaustion, mediated by employees’ experienced guilt. This study underscores the relevance of work–pet family conflict as a distinct form of work–life conflict and highlights the role of guilt as a key emotional driver that contributes to employees’ emotional exhaustion in this context. Hence, organizations can delineate strategies to mitigate work–pet family conflict by offering flexible work arrangements, implementing pet-friendly policies, providing pet care benefits, and fostering a culture that supports work–life balance. These measures can potentially help employees better manage the demands of both work and pet responsibilities.

## Simple Summary

Work–pet family conflict, a novel form of work–life conflict, reflects the growing importance of pets in modern families. Grounded in role theory, this study examined the link between work–pet family conflict and emotional exhaustion, exploring guilt as a mediating factor. Data from 356 pet owners revealed that work–pet family conflict significantly contributes to emotional exhaustion, with guilt acting as a key emotional mechanism. These findings highlight the distinct impact of work–pet family conflict and suggest that organizations can mitigate its effects through flexible work arrangements, pet-friendly policies, and pet care benefits, fostering better work–life balance for employees.

## 1. Introduction

Since the onset of the recent pandemic, the global number of companion animals or pets has increased significantly [1,2]. In fact, families in the U.S., Brazil, the EU, and China account for more than half a billion dogs and cats, with over half of the global population estimated to have a pet at home. For instance, in the U.S., 70% of households owned a pet as of 2021, up from 68% in 2016. Similarly, in 2022, Europe recorded approximately 340 million companion animals, a 27 million increase from 2021 [3]. In Portugal, around 3.1 million companion animals were registered in the Companion Animal Information System (SIAC) that same year, reflecting an increase of 800,000 from 2021 [4]. Across Europe in 2022, cats accounted for 127 million of the companion animals, followed by 104 million dogs, with birds, small mammals, fish, and reptiles in lesser numbers. In Portugal, cats numbered around 1.8 million, while dogs totaled 2.6 million, with birds, small mammals, and reptiles also present in smaller numbers [3]. A similar trend is observed in the United States and China, where dogs outnumber cats.

These numbers reflect a growing interest in pet ownership, with the number of families with pets rising and their family perception as well [5]. Modern families increasingly view pets as “more than just animals”, often considering them as “furry children” [6,7]. Despite these trends, little attention has been given to exploring new forms of work–life conflict related to pet ownership—work–pet family conflict [8]. As many families perceive their pets as important family members, or even as “furry children”, it is likely that they experience work–life conflict when they are unable to attend to pet-related activities or responsibilities [8]. Therefore, investigating this emerging form of work–life conflict is both timely and relevant [9].

Work–pet family conflict occurs when work obligations disrupt pet–family life or interfere with pet-related responsibilities [8]. For example, this conflict may arise when employees are required to work late, leaving their pets alone for extended periods without addressing their basic physiological needs, or when they have to leave their pets due to travel obligations [10]. Similar to work–family conflict, work–pet family conflict can lead to feelings of guilt among pet owners as a reaction to this disruption.

Guilt is an unpleasant emotional state associated with the belief that one’s actions, thoughts, or intentions may be wrong or with the perception that others might view them negatively [11,12]. It is characterized by painful emotions, often accompanied by a sense of responsibility and remorse, in response to specific circumstances [13], such as work–family conflict [9,13,14]. Individuals experiencing guilt tend to blame themselves for perceived shortcomings, whether real or imagined, such as failing to attend to their pets or lacking the energy to engage with them [15]. This type of guilt, known as parental guilt, arises from the competing demands of work and family responsibilities [16]. In the context of work–pet family conflict, guilt might arise when an employee feels they have neglected their pet’s needs due to work demands. For instance, when pet owners come home tired and mentally exhausted, with little energy left to care for their pets—an example of work–pet family conflict—they may feel guilty, believing that their fatigue is harming their relationship with their pets.

Although other emotions, such as frustration or anticipation, may also be relevant to work–pet family conflict, guilt is distinct in that it involves a sense of responsibility or remorse for a perceived wrongdoing or failure, often with moral or ethical implications [11]. Guilt arises when an individual believes they have caused harm or failed to meet expectations, whether toward others or themselves [12]. In contrast, frustration is associated with unmet goals or obstacles, while anticipation pertains to emotions related to future expectations. Each of these emotions influences employees in different ways, shaping their behavior and responses to work–pet family conflict.

In addition to guilt, the interference between work and family responsibilities can serve as an additional source of distress and emotional exhaustion [17,18,19], because individuals expend resources to manage it, such as employing coping strategies to protect their resources and prevent further losses [20,21]. When these strategies fail and employees try to balance job demands with pet–family responsibilities, resource depletion resulting from work–pet family conflict not only triggers negative emotional reactions, such as guilt, but can also lead to distress and emotional exhaustion [20,22]. Emotional exhaustion is the core component of burnout and refers to the depletion of one’s emotional resources [23].

Furthermore, when employees invest significant effort into protecting valuable resources, including relationships with family and pets, and fail to achieve this—especially in the context of work–pet family conflict—they incur additional resource losses that may be essential for coping with daily demands [21]. Therefore, when employees struggle to establish a balance between their job roles and pet–family responsibilities, they are likely to experience work–pet family conflict, which can induce feelings of guilt and contribute to increased emotional exhaustion.

**Figure 1:**
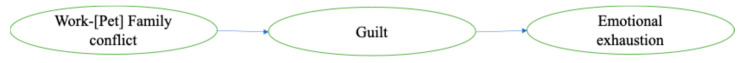
The proposed conceptual model.

Although the relationship between work–family conflict and guilt has been empirically demonstrated [16,17,18], to the best of my knowledge, scarce studies have investigated this relationship within the context of families with pets (see an exception, Kogan et al. [9,13]). As a result, work–pet family conflict has been largely overlooked [8,13]. Therefore, drawing on role theory, this study aims to expand the understanding of work–family conflict by examining its implications for families with pets. Specifically, it explores whether and how work–pet family conflict contributes to emotional exhaustion through the elicitation of guilt (see Figure 1).

This study has three main contributions to this theory. First, it answers the call for studies on work–pet family conflict [8,13] as a domain overlooked that demands empirical exploration due to the increased number of families with pets [1,2,3] and their increasing consideration of them as family members [6,24,25]. Furthermore, examining pets within the context of work–family conflict not only broadens the understanding of their role in organizational life but also underscores the growing significance of incorporating pet–family dynamics into discussions of work–life balance. This exploration encourages further research in this area while raising awareness among practitioners about the unique needs of pet owners in the workplace.

Second, the study extends the existing understanding of work–family barriers by introducing work–pet family conflict as a novel concept. By examining this type of conflict, the research addresses a critical gap in the literature, offering valuable insights into how work-related demands uniquely affect pet owners. Specifically, it highlights how job duties may induce feelings of guilt and emotional exhaustion when they interfere with pet-related responsibilities, thereby creating a sense of imbalance between work and pet–family roles. This expanded focus not only deepens our comprehension of work–family dynamics but also illuminates the distinct emotional consequences faced by pet owners as they navigate the complexities of managing professional obligations alongside pet–family commitments.

Third, the study advances role theory by applying it to the context of families with pets. This application enriches the theoretical framework surrounding work–family conflict by integrating the distinctive dynamics associated with pet ownership. By examining how pet-related responsibilities interact with work demands, the study broadens the scope of role theory, offering a more nuanced understanding of resource depletion and stress in the context of balancing professional and pet–family roles.

This study offers practical implications to guide organizations in making empirically informed decisions regarding the implementation of pet-friendly practices. For example, organizations aiming to reduce employees’ work–pet family conflict can leverage these findings to develop targeted policies. Strategies may include offering flexible work arrangements, implementing pet-friendly workplace initiatives, providing pet care benefits, and fostering a culture that promotes work–life balance. Such measures enable employees to better manage the dual demands of work- and pet-related responsibilities, ultimately enhancing their well-being and productivity.

## 2. Literature Review

### 2.1. The Concept of Work–Pet Family Conflict

Work–family conflict is currently recognized as a significant concern, with various impacts across multiple domains: on a personal level, affecting physical health (e.g., eating habits, physical activity, and physical symptoms) and mental health (e.g., depressive and anxiety symptoms, life satisfaction, happiness, and stress); work-related outcomes (e.g., job satisfaction, motivation, organizational identification, and turnover intentions); and family-related outcomes (e.g., family satisfaction) [26,27,28].

Work–family conflict arises when conflicting role demands from work and family become incompatible and can be manifested in three ways: time-based, behavior-based, and pressure-based conflict [27]. Time-based conflict occurs when time dedicated to one domain, such as working late, limits the time available for family responsibilities. Behavior-based conflict arises when behaviors required in one domain are incompatible with the other; for instance, employees may need to sacrifice family activities to meet work goals. Pressure-based conflict occurs when stress experienced in one domain leads to symptoms such as tension, fatigue, anxiety, depression, apathy, or irritability, which spill over and interfere with the other domain. For example, a stressful workday may produce negative emotions that affect one’s personal life outside of work.

Despite extensive research on work–family conflict, a critical aspect remains largely overlooked—pets [8,9]. As pets are increasingly regarded as family members in modern households [6,24,25], integrating them into discussions on work–family conflict is essential [8,13]. Pet ownership directly affects individuals’ time and behavior at work in various ways [9]. Employees often need to allocate time for pet care activities, such as feeding, walking, or veterinary appointments, which can disrupt work schedules and necessitate adjustments like arriving late, leaving early, or taking breaks during the day to check on their pets. Many pet owners may also prefer flexible or remote work arrangements to meet their pets’ needs, reshaping how they approach and structure their work tasks [8].

For remote workers, pets at home may occasionally disrupt focus due to their needs or behavior, such as barking or requiring attention during meetings. On the positive side, pet ownership often promotes greater routine and responsibility, which can enhance time management and organizational skills, although it may also increase stress when work demands conflict with pet care responsibilities [13]. Additionally, pet ownership can positively influence workplace behavior by reducing stress, improving mood, and fostering social interactions, especially in workplaces with pet-friendly policies, such as allowing pets on-site. However, guilt or worry about leaving pets alone for extended periods can negatively impact an employee’s emotional well-being and productivity.

Recognizing the growing importance of pets to their employees, organizations are increasingly implementing pet-friendly policies, as these practices have demonstrated positive effects on both employees and organizational outcomes [7,29,30,31]. Incorporating pets into the study of work–family conflict provides a more comprehensive understanding of the work–life interface, particularly for Millennial and Generation Z cohorts, who are more likely to view their pets as integral family members [32,33].

Work–pet family conflict refers to the interference of work with pet–family life or pet-related family responsibilities This conflict manifests in scenarios such as employees working long hours, which prevents them from attending to their pets’ needs or leaving pets home alone for extended periods. Similarly, work-related travel often necessitates placing pets in care facilities or relying on friends or family members for their care. These disruptions align with the three dimensions of work–family conflict proposed by Greenhaus and Beutell [27]: time-based conflict, strain-based conflict, and behavior-based conflict. Time-based conflict occurs when work demands, such as extended hours, prevent employees from attending to pet-related responsibilities, such as veterinary appointments. Pressure-based conflict arises when work-induced stress leaves employees emotionally depleted, diminishing their capacity to engage with their pets at home. Behavior-based conflict manifests when work commitments, such as business travel, require employees to entrust their pets to care facilities or rely on family members for assistance [8].

### 2.2. The Relationship Between Work–Pet Family Conflict and Emotional Exhaustion

When employees exert effort to balance job demands with family responsibilities, employees may deplete resources such as time and energy, leading to feelings of distress associated with work–family conflict [16,22,28]. Work–pet family conflict can therefore be understood as a form of resource loss, as individuals must expend additional personal resources to manage competing demands, which may lead to stress and emotional exhaustion [8]. Specifically, when employees attempt to balance work responsibilities with pet–family obligations, they may experience significant personal resource depletion, ultimately resulting in elevate emotional exhaustion levels [8,13,16].

Emotional exhaustion refers to the depletion of one’s emotional resources, resulting in a state of fatigue [23]. It is considered the central dimension of burnout [34,35]. Burnout is a prolonged response to chronic stress characterized by a psychological syndrome that includes emotional exhaustion, depersonalization, and a diminished sense of personal accomplishment. This condition has been linked to work–family conflict, with several studies demonstrating that it frequently arises as a consequence of negative work–family interactions [18,36,37,38], including work–pet family conflict.

For instance, research on work–pet family conflict has gained momentum, with studies by Kogan et al. [13] and Applebaum and Zsembik [9] offering valuable insights. Kogan et al. [13] explored the challenges faced by pet owners during the COVID-19 pandemic, highlighting how increased remote work brought both benefits and stressors for individuals balancing work responsibilities with pet care. Their findings revealed that, while pets often provided emotional support and stress relief, they also introduced unique demands, such as disruptions during work hours, that intensified work–pet family conflict. Similarly, Applebaum and Zsembik [9] examined the broader implications of pet ownership on family dynamics and work–life balance, emphasizing that pets are integral to family systems and can contribute to time-based and strain-based conflicts when work obligations interfere with pet care responsibilities. Both studies underscored the importance of work–pet family conflict for employees’ well-being and quality of life.

### 2.3. The Mediating Role of Guilt

As articulated by role theory, when employees invest considerable effort in protecting valuable resources—such as relationships with family and pets—and are unable to achieve this, particularly in the context of work–pet family conflict, they incur additional resource losses that are essential for coping with daily demands [21]. Consequently, when employees struggle to balance their job roles with pet–family responsibilities, they are likely to experience work–pet family conflict, which may lead to elevated levels of emotional exhaustion. This increase in emotional exhaustion can be partly attributed to the feelings of guilt that arise from the experience of work–pet family conflict [11,13,14].

While other emotions, such as frustration or anticipation, may also influence work–pet family conflict, guilt stands out due to its distinct connection to moral or ethical considerations. Guilt arises when individuals perceive that they have caused harm or failed to meet expectations, whether directed toward others or themselves [12]. In contrast, frustration is tied to the experience of unmet goals or obstacles, whereas anticipation relates to emotions associated with future expectations. Each of these emotions impacts employees differently, shaping their behaviors and responses to work–pet family conflict in unique ways.

Guilt is an unpleasant emotional state associated with the belief that one’s actions, thoughts, or intentions may be wrong or with the perception that others might view them negatively [11,12]. It is characterized by painful emotions, often accompanied by a sense of responsibility and remorse in response to specific circumstances [13], such as failing to care for family pets [14].

In the context of work–family conflict, guilt has been referred to as work–family guilt [39,40]. Work–family guilt is defined as the emotional discomfort stemming from a perceived discrepancy between one’s desired and actual participation in both work and family roles [41]. This type of guilt often emerges when work obligations encroach on family life [42], including pet–family responsibilities or when individuals struggle to balance their professional and personal commitments [43,44]. Consequently, work–family guilt is frequently understood as a response to the difficult choices individuals face when navigating competing demands between their work and family roles [45,46].

Individuals experiencing this kind of guilt tend to blame themselves for perceived shortcomings, whether real or imagined, such as neglecting to attend to their pets or lacking the energy to engage with them [15]. This type of guilt, often referred to as parental guilt, arises from the competing demands of work and family responsibilities [16,41,42]. For instance, when pet owners return home fatigued and mentally exhausted, with little energy left to care for their pets—an illustration of work–pet family conflict—they may feel guilty, believing that their fatigue negatively impacts their relationship with their pets.

Experiencing guilt in response to work–pet family conflict can lead to emotional exhaustion, as the negative emotional state associated with guilt can significantly drain an individual’s psychological resources [39,40]. When employees find themselves torn between their professional responsibilities and their commitments to their pets, they may feel that they are failing to meet the expectations they have for themselves as pet parents [46]. This internal conflict can create a cycle of guilt, where individuals blame themselves for not being present or attentive enough to their pets, which can exacerbate feelings of inadequacy and distress and deplete their resources even more [41,47].

As guilt intensifies, it may manifest in various ways, such as increased anxiety, irritability, and a pervasive sense of remorse [12,13]. These emotions can further deplete the individual’s emotional resources, making it increasingly difficult to cope with daily demands both at work and at home [14,15]. The emotional toll of guilt may also hinder one’s ability to engage in restorative activities, such as spending quality time with their pets, which could otherwise alleviate stress and foster well-being [9,13].

Moreover, the ongoing experience of guilt can lead to a diminished capacity for emotional regulation, as individuals may become more reactive to stressors, both at work and within their pet–family dynamics [12,44,45]. Over time, this emotional turmoil can culminate in emotional exhaustion, characterized by feelings of fatigue, detachment, and a reduced sense of accomplishment [48]. As emotional exhaustion sets in, employees may struggle to maintain productivity at work and may also find it challenging to engage meaningfully with their pets, creating a detrimental cycle that reinforces both work–pet family conflict and emotional fatigue [49,50].

Thus, the connection between guilt and emotional exhaustion emphasizes the necessity of addressing work–pet family conflict, which serves as a significant source of resource depletion for pet owners. This depletion can impair their ability to effectively manage daily demands and responsibilities, ultimately contributing to their emotional exhaustion. Thus, relying on role theory, the following hypothesis was proposed:

**Hypothesis** **1.** *Work–pet family conflict is positively associated with emotional exhaustion via guilt.*

## 3. Methods

### 3.1. Participants and Procedure

The Ethics Committee of the first author’s university approved the study before it started.

Participants were part of the researchers’ professional network and were asked to participate in a study about pet-friendly work environments via email. They were thoroughly informed about the nature and study’s goal, and the confidentiality and anonymity of the data were warranted. Adequate information was provided about the demands that the project would place on them in terms of time and activities required from the respondents, as well as disclosure of confidential information. Respondents were also informed that they were free to participate, to decline to participate, or to withdraw from the research at any time. Since the questionnaires were shared online (via email), the above-mentioned information was provided in the cover letter of the questionnaire.

Two waves of data were collected to mitigate the potential issue of common method variance. Additionally, several precautionary measures were implemented to reduce the risk of common method bias (CMB) [51]. These measures included randomizing the order of items and incorporating screening questions in the questionnaires. Data collection took place from May to September 2024.

In the first phase (Time 1), 540 surveys were distributed, which included measures of work–pet family conflict and socio-demographic information. A total of 487 responses were received, resulting in a high response rate of 90.18%. In the second phase (Time 2), one week later, questionnaires assessing guilt and emotional exhaustion were sent to the 487 participants who completed the initial survey. At this stage, 378 completed surveys were collected, reflecting a response rate of 70%. After excluding invalid responses (e.g., surveys completed in under two minutes or with perfunctory answers), 356 valid responses remained, producing an overall response rate of 65.92%.

The sample size was calculated using GPower statistical power analysis software *(G*Power 3.1.9.7; Kiel University, Germany) for a linear multiple regression model with three predictors (i.e., the three socialization tactics). The input parameters were as follows: statistical test = *t*-tests: linear multiple regression; effect size *f*^2^ = 0.15; α error probability = 0.05; power (1—β error probability) = 0.95; and number of predictors = 2. Based on these parameters, the required sample size was determined to be 74 participants. Therefore, the sample of 356 was considered sufficient for testing the model.

The final sample comprised 356 pet owners, with an average age of 30.88 years (SD = 10.74) and an average organizational tenure of 10.33 years (SD = 10.97). Of the participants, 46% were female. In terms of educational background, over half (70.1%) held at least a bachelor’s degree. The participants had an average of 4.46 pets (SD = 2.12), and they had been pet owners for an average of 8.52 years (SD = 2.97). Most respondents reported having dogs (80.05%), followed by cats (16.29%), with all pets living within the family home.

### 3.2. Measures

#### 3.2.1. Work–Pet Family Conflict (T1)

We used the work–pet family boundaries scale [52]. Three items were used to assess time-based conflict (“I have to miss activities with my pets (or engage in fewer activities with them) due to the amount of time I must spend on work responsibilities.”), tension-based conflict (“I am often so emotionally drained when I get home from work that it prevents me from contributing to my pets.”), and behavioral-based conflict (“The behaviors I perform that make me effective at work do not help me to be a better pet parent.”). The items were answered on a 5-point Likert scale (1—Strongly Disagree; 5—Strongly Agree) (α = 0.77; ω = 0.82).

#### 3.2.2. Guilt (Time 2)

To measure guilt, five items from PANAS [53] (e.g., “guilt”) were answered on a five-point Likert scale (1—never; 5—always); (α = 0.90; ω = 0.90).

#### 3.2.3. Emotional Exhaustion (Time 2)

Nine items from the MBI developed by Maslach et al. [35] were used to measure emotional exhaustion (e.g., “I feel burned out from my work.”). Participants reported the frequency with which they felt emotionally exhausted on a 5-point Likert scale (1—never; 5—daily) (α = 0.82; ω = 0.82).

### 3.3. Control Variables

We used participants’ sex and age as controls. We used sex as a control, because some studies have shown that women tend to be more sensitive and empathetic to animals than men [48]. Hence, sex differences could influence both mediator and the criterion variable. Furthermore, age could also account for influences on emotional exhaustion, as there have been identified differences in the way older and younger experiences affect and their subsequent levels of well-being [50].

### 3.4. Data Analysis

In the proposed mediating model (see Figure 1), there were three types of variables: (1) predictor (work–pet family conflict); (2) one criterion variable (emotional exhaustion); and (3) one mediator (guilt). SPSS 28.0 and the software JASP (version 0.14.1) were used to test the proposed research model. First, descriptive analysis was conducted to calculate the mean and standard deviation for each variable. Second, correlational analyses were performed to examine whether work modality was associated with the mediator and the criterion variables. Third, the measurement model’s goodness of fit was evaluated. In this regard, we found that the Root Mean Square Error of Approximation (RMSEA) < 0.08, Standardized Root Mean Squared Residual (SRMR) < 0.08, Comparative Fit Index (CFI) > 0.90, and Tucker–Lewis Index (TLI) > 0.90 evidenced a good fit [54].

## 4. Results

### 4.1. Common Method Bias and Multicollinearity Issues

To understand the presence of common method bias in the study, we followed some recommendations. First, we performed Harman’s single factor test to check for common method bias. The findings showed that the first factor only accounted for 22.25% of the total explained variance; hence, the common method bias was not a serious issue.

Second, we performed three confirmatory factor analyses (CFA) to test the independence of the variables under study. To assess the adequacy of the model and compare it with other reasonable alternative models, we analyzed diverse fit indices, namely CFI, TLI, SRMR, and RMSEA [55]. Model 1 was the hypothetical three-factor model comprising separate scales for work–pet family conflict, guilt, and emotional exhaustion. In contrast, Model 2 tested a two-factor structure, combining guilt and emotional exhaustion into a single factor due to their conceptual and emotional similarity, along with work–pet family conflict loaded onto another factor. Model 3 was a single-factor solution where all items were loaded onto a single latent factor. Table 1 shows that the three-factor model (Model 1) provided the best fit to the data (χ^2^/df = 1.61, *p* < 0.001, CFI = 0.98, TLI = 0.97, SRMR = 0.05, and RMSEA = 0.05 CI 95% [0.01, 0.08]), and all other alternative models showed a poorer fit. These results, along with reliability indices measured through Cronbach’s alpha in all measurement scales, demonstrated the discriminant and convergent validity of the study; therefore, we proceeded with testing the two hypotheses.

### 4.2. Descriptive Statistics

Table 2 shows the correlations between the variables, as well as their mean values and standard deviations. The results also showed that all variables were significantly correlated with each other in the expected direction.

The result of convergent validity, which measures how the indicators of the latent construct correlate, revealed that the values of Average Variance Extracted (AVE) for all latent constructs in the study were above 0.5. Additionally, the AVE for each construct was evaluated concerning its correlation with other constructs, and the AVE value was found to be higher than the correlation of the construct with other constructs, thus supporting convergent validity.

Discriminant validity indicates the extent to which the constructs in a study are distinct from one another. It demonstrates that each construct measures a unique concept and is not overly correlated with other constructs. In the context of a study, strong discriminant validity ensures that the scales used to measure different variables, such as work–pet family conflict, guilt, and emotional exhaustion, are capturing separate dimensions of the phenomenon rather than overlapping or redundant aspects. This validity is essential for drawing accurate conclusions about the relationships between constructs and supports the theoretical framework underpinning the research. The square roots of the Average Variance Extracted (AVE) indicated by the diagonal value of each latent variables were all greater than the correlations of each variable. Maximum Shared Variance (MSV) was also analyzed; the results of MSV showed that it was lower than AVE for all constructs; thus, discriminant validity was supported. In this way, the reliability, convergent validity, and discriminant validity of the study were confirmed. Based on the validity of the study instrument, the study hypotheses were analyzed.

### 4.3. Hypotheses Testing

**Figure 2:**
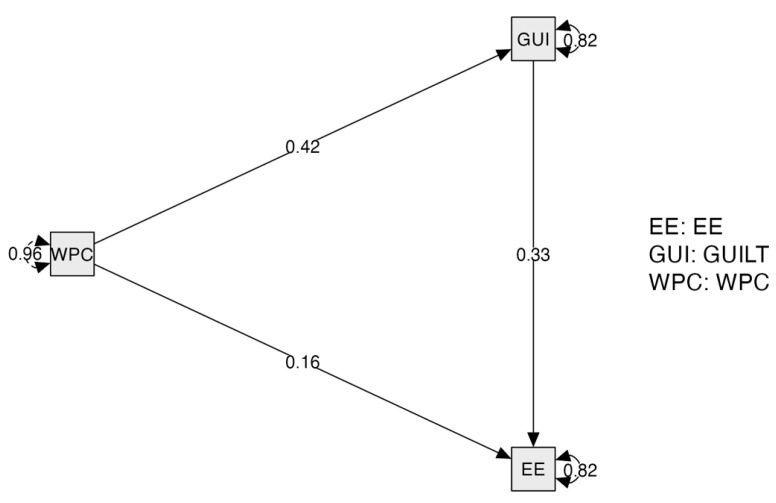
Path coefficients.

The structural equation model fit the data well: χ^2^_(df)_ = 1.50, *p* < 0.01, CFI = 0.99, TLI = 0.99, RMSEA = 0.05, 90% CI [0.00; 0.13]), and SRMR = 0.04. The standardized path coefficients between the variables are presented in Figure 2.

The test of the indirect effect showed that experienced guilt significantly mediated the relationship between work–pet family conflict and emotional exhaustion (β = 0.14; *p* < 0.001; 95% CI [0.04; 0.24]). Moreover, the results evidenced a direct significant effect from work–pet family conflict on both guilt (β = 0.42, *p* < 0.001) and emotional exhaustion (β = 0.16, *p* < 0.05). The model explained 18% of the variance in emotional exhaustion (R^2^ = 0.18). Thus, hypothesis 1 was supported by the data (see Table 3).

## 5. Discussion

This study examines the relationship between work–pet family conflict and emotional exhaustion, drawing on role theory. We propose that work–pet family conflict leads to feelings of guilt, which, in turn, contributes to increased emotional exhaustion. Similar to work–family conflict, work–pet family conflict appears to produce comparable effects [13,16]. Our findings suggest that, when work obligations interfere with pet–family responsibilities, pet owners experience heightened levels of emotional exhaustion, largely due to the guilt they feel over neglecting their pets as a result of their work commitments. The resultant guilt may occur because pet owners may perceive their inability to care for their pets as a personal failure [9]. Consequently, the emotional toll of managing both work and pet–family roles can contribute to emotional exhaustion.

### 5.1. Theoretical Implications

This study highlights the importance of recognizing work–pet family conflict as a genuine source of resource depletion and emotional exhaustion. By focusing on the emotional consequences of this conflict, particularly guilt and emotional exhaustion, it offers new insights into both the theoretical and practical dimensions of balancing work and personal responsibilities in the context of pet ownership.

Given the societal shifts in pet ownership and how pets are increasingly viewed as family members [32,33,55,56], it is crucial to incorporate pets into discussions of work–family dynamics [8]. Understanding how pet owners experience work–family conflict, especially in relation to their pets, is essential [8,13]. This study addresses the gap in research on work–pet family conflict, responding to calls for further empirical exploration of this overlooked domain [56].

Second, this study expands the current understanding of work–family challenges by introducing work–pet family conflict as a novel concept [57]. By examining this specific form of conflict, the research addresses an important gap in the literature, providing valuable insights into how work-related demands uniquely impact pet owners [8,9]. Specifically, it underscores how job responsibilities can evoke feelings of guilt and emotional exhaustion when they interfere with pet-related duties, leading to a sense of imbalance between work and pet–family roles [58].

This extended focus deepens the comprehension of work–family dynamics by illuminating the distinct emotional repercussions that pet owners face as they manage both professional and pet–family obligations. In doing so, the study reveals how the emotional toll of work–pet family conflict mirrors traditional work–family conflict while also presenting unique challenges specific to pet owners. This highlights the need for a broader, more inclusive approach to understanding work–life balance that accounts for the growing role of pets in family life.

Third, this study advances role theory by applying it to the context of families with pets, thereby extending its utility in understanding work–family conflict. This application enriches the theoretical framework by incorporating the unique dynamics of pet ownership into the discussion of resource depletion and stress. By exploring how pet-related responsibilities interact with work demands, the study broadens the scope of role theory, offering a more nuanced perspective on how individuals manage resource loss in the face of competing professional and pet–family roles.

Work–pet family conflict refers to the tension that arises when work responsibilities interfere with pet-related duties, creating a scenario where employees must juggle competing demands. The findings highlight that work–pet family conflict triggers guilt, which acts as a key mediator in the relationship between this conflict and emotional exhaustion. Guilt, an unpleasant emotional state characterized by a sense of failure or responsibility, arises when individuals perceive that they are neglecting their pets due to work commitments [11,12,14]. This feeling of guilt intensifies the emotional strain, leading to emotional exhaustion, a key component of burnout [23]. Emotional exhaustion reflects the depletion of emotional resources and is commonly associated with chronic stress, including stress arising from imbalances between personal and professional roles [17,19,28]. Our findings indicate that, similar to the well-documented work–family conflict [16,27,38,39], work–pet family conflict can have comparable adverse effects [9,57,58]. Pet owners who experience this form of conflict report higher levels of emotional exhaustion, largely because they feel guilty for not fulfilling their pet-related responsibilities. This guilt amplifies their emotional fatigue, as they may view their inability to care for their pets as a personal failure. Consequently, the emotional toll of managing both work and pet–family roles can contribute to overall emotional exhaustion.

This extension of role theory deepens our understanding of how resource depletion occurs not only in traditional family settings but also in pet-owning households. It reveals that the emotional toll associated with managing both work- and pet-related obligations can result in significant resource loss—time, energy, and emotional capacity—ultimately leading to emotional exhaustion. As such, the study contributes to a more comprehensive understanding of the stressors that influence work–life balance, emphasizing the need to consider the role of pets as an integral part of family life in both theoretical and practical contexts.

### 5.2. Practical Implications

Organizations can take several steps to mitigate work–pet family conflict and help employees balance the demands of both work- and pet-related responsibilities. For instance, organizations can implement pet-friendly policies or offer flexible work arrangements to employees facing challenges in managing their work and pet–family responsibilities. Flexible work options, such as flexible hours or hybrid work, allow employees to structure their schedules to accommodate both work duties and pet care. By offering the ability to work from home or adjust schedules, organizations can reduce time-based conflicts and alleviate stress associated with rigid work hours.

Contemporary generations, especially younger employees, increasingly prioritize flexible work options, such as hybrid work and work–life balance, in contrast to older generations, who traditionally value job stability and career progression within organizations. Younger employees, who often consider their pets as family members, may experience less work–pet family conflict when offered flexible work arrangements. Hybrid work offers increased flexibility, allowing employees to better manage both their work responsibilities and pet-related tasks, such as feeding, walking, and providing emotional care. This flexibility can reduce time-based conflicts by enabling employees to arrange their schedules to accommodate pet care without compromising work obligations. Additionally, hybrid work can help employees care for pets that require extra attention, such as during illness. This approach could enhance job satisfaction, overall employee engagement, and work–life integration.

Introducing pet-friendly workplace policies, such as allowing employees to bring pets to the office or hosting “pet days”, can help integrate work and pet responsibilities, reducing stress and improving morale. Offering pet care benefits, such as pet insurance or subsidies for pet-related services, can alleviate the financial burden of pet ownership, particularly during illness or emergencies. By promoting a culture that prioritizes work–life balance, organizations can support employees in managing stress and preventing burnout. Encouraging regular breaks, wellness programs, and promoting access to mental health resources are also important for maintaining a healthy balance between work and personal life. Employee assistance programs (EAPs) that offer counseling or resources related to family life, including pet care, can help employees cope with the emotional challenges of work–pet family conflict.

Moreover, organizations can create awareness and provide training for managers and employees about work–pet family conflict, helping them implement strategies to manage potential challenges and establish clear boundaries between work and personal life. Finally, fostering an organizational culture that values empathy, support, and open communication can reduce stigma and help employees manage their well-being. By adopting these strategies, organizations can help alleviate work–pet family conflict, ultimately improving employee well-being, productivity, and engagement.

Technological advancements, such as pet care apps and virtual monitoring tools, are likely to have a significant impact on the dynamics of work–pet family conflict. These technologies can either alleviate or intensify conflict, depending on how they are incorporated into employees’ work and personal lives. On the one hand, tools like pet care apps, automated feeding systems, and smart pet cameras can help employees manage their pets’ needs more efficiently. For instance, pet owners can remotely monitor their pets’ activities on days they are working in person, ensure they are fed, or provide comfort through interactive toys or virtual communication. This could reduce time-based conflict, and, in part, alleviate feelings of guilt about leaving pets alone, thereby easing the emotional burden of balancing work and pet responsibilities.

Additionally, virtual tools that allow employees to check in on their pets during breaks or interact with them remotely could enhance work–life integration, especially for those who are away from home for extended periods. The ability to monitor pets while working long hours or traveling for business may help employees feel more connected to their pets, reducing stress and mitigating work–pet family conflict.

### 5.3. Limitations and Future Directions

This study presents several limitations that warrant consideration. Firstly, although it employed a two-wave design, it does not allow for the observation of fluctuations in work–pet family conflict, guilt, and emotional exhaustion over time. This is a significant limitation, as previous research indicates that affective indicators may vary across different time points [48,49]. Future studies could address this limitation by adopting a longitudinal or daily diary approach to capture dynamic changes in these variables over time. A longitudinal study has the potential to allow for the observation of changes and patterns in these variables as they evolve. Unlike cross-sectional studies or two-wave studies, which capture data at a single point in time, longitudinal studies track individuals over an extended period, enabling researchers to observe how guilt and emotional exhaustion fluctuate in response to changing work conditions, personal circumstances, or other external factors. For example, guilt related to work–pet family conflict may not be a static emotion; it could vary, depending on the intensity and frequency of work demands, the availability of pet care resources, or changes in work arrangements (e.g., transitioning to remote or hybrid work). Similarly, emotional exhaustion may develop or dissipate over time, influenced by cumulative stressors, organizational support, or the implementation of coping strategies. By capturing these fluctuations, a longitudinal study would provide a clearer understanding of the causal relationships between work–pet family conflict, guilt, and emotional exhaustion, revealing how they reinforce or mitigate each other over time.

Secondly, the reliance on self-reported measures may introduce common method bias, potentially influencing the accuracy of the relationships between constructs [51]. However, several precautionary steps were taken, including confirmatory factor analysis, internal consistency checks, and Harman’s single-factor test, all of which suggest that common method bias does not pose a significant issue in this study.

Thirdly, the reliance on a professional network for sampling introduces potential concerns about selection bias, which could affect the generalizability of the findings. Future studies should aim to diversify sampling methods to include participants from a broader range of backgrounds and contexts.

In addition, in daily life, various pressures contribute to frustrations and guilt, including excessive workload, low income, chronic illnesses, marital frustrations, and a poor social life [9]. On the other hand, some activities and events can help alleviate guilt, such as engaging in religious activities, leisure, hobbies, or other restorative practices [13]. Similarly, compensatory outputs of guilt, such as participation in religious activities, political engagement, or involvement in charitable organizations, act as escape channels that could provide emotional relief. These elements, however, are not considered in the current model. Furthermore, this study seems focused on problems, feelings, and perceptions of guilt and emotional activity specifically arising from daily conflicts between work and pet–family responsibilities. While this approach sheds light on a particular aspect of the issue, it overlooks other important dynamics that could be equally relevant. As such, future studies could expand on this by considering additional inputs—such as broader socioeconomic factors, health-related issues, or relationship dynamics—and outputs, such as coping mechanisms or adaptive behaviors beyond work–pet family conflict. Such an expanded framework would provide a more holistic understanding of the complexities surrounding guilt, emotional exhaustion, and their associated factors, offering richer insights into the interplay between the personal, work, and family domains.

Furthermore, the mediation model presented, which focuses on guilt as a central emotional response, is compelling; however, future research could benefit from exploring other affective responses to work–pet family conflict. Emotions such as frustration, anxiety, or even feelings of inadequacy may also play a significant role in shaping outcomes like emotional exhaustion, well-being, or coping strategies. These affective responses could provide a more nuanced understanding of how individuals manage the competing demands of work- and pet-related responsibilities. By broadening the scope to include a wider range of emotions, future studies could uncover additional pathways through which work–pet family conflict influences both psychological and behavioral outcomes, contributing to a richer and more comprehensive framework.

Lastly, another limitation is the absence of an analysis of participants’ attitudes toward their pets, which could play a role in how they experience work–pet family conflict [8]. Future research should incorporate measures of pet-related attitudes or levels of attachment between pet owners and their pets to gain deeper insights into these dynamics.

## 6. Conclusions

Our findings indicate that, when work obligations conflict with pet–family responsibilities, pet owners tend to experience increased levels of emotional exhaustion. This emotional strain appears to be primarily driven by the guilt associated with neglecting their pets due to work demands. This guilt, arising from perceived shortcomings in fulfilling pet-related responsibilities, serves as a key mechanism linking work–pet family conflict to emotional exhaustion.

## Figures and Tables

**Table 1:** Confirmatory factorial analysis results.

Models	χ^2^/df	CFI	TLI	RMSEA	SRMR
Model 1	1.61	0.98	0.97	0.05	0.05
Model 2	7.71	0.76	0.64	0.18	0.09
Model 3	10.38	0.65	0.51	0.21	0.11

**Table 2:** Descriptive statistics, correlations, and reliability (Study 2).

Variables	M	SD	CR	AVE	MSV	1	2	3	4
1. WPFC	2.90 ^1^	0.98	0.87	0.68	0.17	(0.82]	[0.77]		
2. Guilt	2.35 ^1^	0.99	0.92	0.71	0.18	0.41 **	(0.84)	[0.90]	
3. EE	3.32 ^1^	0.94	0.89	0.74	0.18	0.29 **	0.42 **	(0.86)	[0.82]
4. Age	30.88	10.74	-	-	-	−0.12	−0.17 *	0.01	-
5. Sex ^2^	-	-	-	-	-	0.01	−0.02	−0.13	0.06

Note. *N* = 356; * *p* < 0.05 and ** *p* < 0.001. ^1^ Scale from 1 to 5. ^2^ Sex code: 1—female; 2—male. The square roots from the Average Variance Extracted (AVE) are in brackets. M = Mean; SD = Standard deviation; AVE = Average Variance Extracted; MSV = Maximum Shared Variance. CR = Composite reliability. Cronbach alphas are in [ ]. WPFC = work–pet family conflict. EE = emotional exhaustion.

**Table 3:** Direct and indirect effects.

Indirect Effects						Estimate	*p*	CI 95% LLCI	ULCI
WPFC		→	Guilt	→	Emotional exhaustion	0.14 **	0.0004	0.04	0.24
Direct effects									
WPFC	→		Guilt			0.42 **	<0.01	0.15	0.51
WPFC	→		Emotional exhaustion			0.16 *	<0.05	0.00	0.35
Total effects									
WPFC	→		Emotional exhaustion			0.30 **	<0.001	0.12	0.48

Note. *N* = 356; * *p* < 0.05 and ** *p* < 0.001. WPFC = work–pet family conflict.

## Data Availability

Data will be made available upon reasonable request from the author (analjsilva@gmail.com).
